# Physicochemical and Functional Characterization of Pearl Millet-Based Probiotic Beverage for Antiaging Potential in *Caenorhabditis elegans*

**DOI:** 10.3390/foods14203460

**Published:** 2025-10-10

**Authors:** Nova Henna Jemimah Kaila, Prakash M. Halami, Chethana Ramakrishna, Mamatha Singanahalli Shivaramu, Muthukumar Serva Peddha

**Affiliations:** 1 Department of Biochemistry, CSIR—Central Food Technological Research Institute (CFTRI), Mysore 570020, Karnataka, India; njems768@gmail.com; 2 Department of Microbiology and Fermentation Technology, CSIR—Central Food Technological Research Institute (CFTRI), Mysore 570020, Karnataka, India; 3 Department of Traditional Foods and Applied Sciences, CSIR—Central Food Technological Research Institute (CFTRI), Mysore 570020, Karnataka, India; 4 Department of Food Protectants and Infestation Control, CSIR—Central Food Technological Research Institute (CFTRI), Mysore 570020, Karnataka, India

**Keywords:** antiaging, *C. elegans*, pearl millet, polyphenols, oxidative stress, inflammation

## Abstract

Probiotics like *Lactobacillus* sp. are extensively studied for their beneficial host interactions, including the gut–brain axis, anti-inflammatory effects, immune system interactions, restoration of gut dysbiosis, and anti-aging effects. In the current study, pearl millet was fermented with *Lactobacillus plantarum* strains DHCU 70 and MCC 5231, which enhanced the nutritional, bioactive, and functional properties of derived probiotic beverages. Compared to unfermented controls, fermented beverages exhibited increased protein content and vitamins B_1_, B_2,_ and B_3_, with decreased carbohydrate and dietary fiber levels. The probiotics have maintained viability exceeding 12 log CFU/mL and showed resistance to harsh gastrointestinal conditions. Fermentation increased total phenolic content from 13.38 ± 0.40 mg GAE/100 g to 42.10 ± 2.65 mg GAE/100 g (LPDB) and 47.76 ± 1.37 mg GAE/100 g (LPMB) and total flavonoid content from 13.01 ± 1.18 mg QE/100 g to 23.12 ± 2.73 mg QE/100 g and 24.21 ± 0.98 mg QE/100 g, respectively. Antioxidant assays showed DPPH radical scavenging improved by 37%, ferrous ion chelation rose from 71.69 ± 0.09 mg TE/100 g to 91.45 ± 0.006 mg TE/100 g, ABTS scavenging increased from 71.62 mg TE/100 g to 82.51 ± 0.04 mg TE/100 g (LPDB) and 89.74 ± 0.04 mg TE/100 g (LPMB) and superoxide radical inhibition rose from 51.40 ± 0.98% to 81.77 ± 0.03% (LPDB) and 79.92 ± 0.02% (LPMB). In the in vivo model, *Caenorhabditis elegans*, fermented beverage treatments significantly improved health-span parameters like head-swing frequency (13.51% increase), body bend frequency (8.41% increase), pharyngeal pumping (8.15% increase) with reduced lipofuscin accumulation and intracellular reactive oxygen species while median lifespan extended beyond 24 days versus 14–16 days in controls (*p* < 0.05). Gompertz mortality modeling revealed a significant decrease in the aging rate parameter, indicating systemic mitigation of stress-induced physiological decline. These combined nutritional, bioactive, and in vivo longevity results underscore the potential of *L. plantarum*-fermented pearl millet beverages as functional nutraceuticals that target oxidative stress and promote healthy aging.

## 1. Introduction

Aging is a time-dependent functional and structural degression of an organism, which is closely studied to be accelerated by free radical production, which induces oxidative stress and the accumulation of which causes damage to cellular macromolecules (lipids, DNA, and proteins) [1]. The proposed oxidation-inflammatory theory of aging or oxi-inflammaging integrates the role of oxidative stress and inflammation as a vicious cycle, which are the primary drivers of aging, leading to acceleration of cellular and tissue damage. This leads to adverse conditions associated with cardiovascular diseases, chronic kidney disorder, diabetes, metabolic syndrome, chronic obstructive pulmonary disease, and neural disorders, all of which are age-dependent [2,3].

Diet is one of the two dominant environmental factors that was studied to influence the pace of aging. Dietary compounds like polyphenols, flavonoids, vitamins, and fiber were studied to alleviate oxidative stress and inflammation, thereby enhancing longevity across different in vitro and in vivo models. Millets contain bioactive compounds like polyphenols, organic acids, short chain fatty acids and free saccharides, which were studied to enhance endogenous antioxidant enzymes and modulate inflammatory pathways [4], scavenge free radicals and protect cellular components from oxidative damage [5], reduce inflammation by NF-kB, support gut barrier function [6]. Millets are studied for their antioxidant properties of phenolic extracts from seven millet varieties [kodo, finger (Ravi), finger (local), proso, foxtail, little, and pearl] on HT-29 cells. All varieties used in this study showed a significant reduction in lipid peroxidation in liposomes, singlet oxygen quenching, and DNA scission inhibition to varying degrees [7]. Furthermore, the cytoprotective effects of various types of millet phenolics, including catechin, ferulic acid, vanillic acid, and resveratrol, were investigated, and it was observed that they exhibit preventive effects on protein and human erythrocyte peroxidation [8].

Literature also shows that probiotics, specifically *Lactobacillus plantarum*, not only enhance the nutritional and bioactive quality of foods, but when administered in effective viability, induce inter-host physiological benefits through interactions like gut–brain axis, cell communication, immune system interaction, pathogen interference, restoration of gut dysbiosis, and antiaging. In the specific context of antiaging, probiotics termed as ‘gerobiotics’ [9] are studied to activate Nrf2 pathway, increasing the expression of antioxidant genes, thereby alleviating oxidative stress which is one of the dominant contributors for aging. Anti-aging refers to interventions targeting cellular and molecular hallmarks of aging to preserve physiological function, whereas longevity denotes quantitatively measured increases in organismal lifespan, assessed by mean and median survival metrics. Also, Salazar et al. (2025) report that they generate metabolites that effectively scavenge reactive oxygen species and enhance mitochondrial function [10]. These strains were also reported to inhibit TLR4 and NF-kB pathways, leading to a reduction in systemic inflammation [11].

The present investigation employed two well-characterized *Lactiplantibacillus plantarum* strains with documented safety profiles and probiotic functionality. *L. plantarum* MCC5231 has undergone a comprehensive safety evaluation, including acute and subacute toxicity assessments, demonstrating non-pathogenic characteristics with confirmed gastrointestinal tract persistence and colonization capacity [12]. The strain exhibits robust tolerance to gastric acidity (pH 2.0) and bile salts (0.3% oxgall), essential prerequisites for probiotic efficacy. Similarly, *L. plantarum* DHCU70, isolated from traditional Indian fermented dairy products, possesses verified probiotic marker genes including those encoding adhesion proteins, bacteriocin production pathways, and stress response mechanisms [13]. 

*C elegans* is a highly suitable model for aging research due to its short and invariant lifespan, ease of maintenance, and low cost, enabling rapid and statistically robust lifespan assay with large populations [14]. The nematode shares homologs for about two-thirds of human disease genes and exhibits clear, quantifiable age-dependent physiological and molecular changes relevant to human aging, including alterations in tissue integrity, motility, stress response, and gene expression [15]. In this in vivo model, millet brain peptides at 12.50 µg/mL have been shown to prolong the lifespan by reducing lipid oxidation, oxidative stress, and lipofuscin levels [16]. Probiotics of the genus such as *Lactobacillus, Bacillus,* and *Pediococcus* were studied to enhance lifespan, in which fermented cereal-origin gerobiotic cocktails were proven to extend the mean lifespan of *C. elegans* by 46.20 to 53.10%, respectively [17]. Despite studies showing that fermentation enhances nutritional value, bioactive value, and antioxidant capacity, millets fermented with probiotics have not been studied for their in vivo ROS scavenging and inflammation alleviation, all pointing toward antiaging, which has been carried out in this study. Our previous work showed pearl millet to contain polyphenols and higher phenolic (4.11 mg GAE/g) and flavonoid content (100.33 mg CE/g), along with higher antioxidant and anti-inflammatory potential [18]. The present study explored the synergy between probiotics and pearl millet, simultaneously observing antioxidant, anti-inflammatory, ROS scavenging potential, and lifespan in vivo.

## 2. Materials

### 2.1. Chemicals and Reagents

2,2-azinobis-(3-ethylbenzothiazoline-6-sulfonic acid) diammonium salt (ABTS), 2,2-diphenyl-1-picrylhydrazyl (DPPH), 2,7-dichlorodihydrofluorescein diacetate (DCFH-DA), FUdR (5-fluoro-2’-deoxyuridine) juglone, lysozyme, amphotericin B, bile, bile salts, carbenicillin, catechin, nicotinamide adenine dinucleotide (NADH), nitro blue tetrazolium, O-phenanthroline, phenazine methosulfate, de Man Rogosa and Sharpe (MRS) agar, de Man Rogosa and Sharpe (MRS) broth, ferulic acid, salicylic acid, S-basal media, S-complete media, hydrochloric acid (HCl), 10% sodium hydroxide (NaOH), ethanol, ferric chloride, ferrous sulfate (FeSO_4_), hydrogen peroxide (H_2_O_2_), methanol, phosphoric acid, potassium citrate, potassium ferricyanide, potassium persulfate, sodium hydroxide, sodium phosphate buffer, Whatman filter paper (11 μm) were procured from Sigma Aldrich, Bangalore, India, trichloroacetic acid, phosphate-buffered saline (PBS), N-(1-naphthyl) ethylene diamine dihydrochloride were procured from Juniper Lifesciences, Bangalore, India).

### 2.2. Ingredients for Beverage

Pearl millet and sweetened cocoa powder used were procured from Loyal World Complex, Vani Vilas Mohalla, Mysore, Karnataka, India. The probiotic strains, *L. plantarum* (DHCU 70) and *Lactiplantibacillus plantarum* MCC 5231, were obtained from the Department of Microbiology and Fermentation Technology, CSIR-CFTRI, Mysuru, Karnataka, India. The selected strains were propagated and incubated for 24 h in sterile MRS broth at 37 °C and stored for subsequent use at 4 °C.

### 2.3. Formulation of Beverage

Based on preliminary studies, pearl millet was thermally processed (121 °C for 15 min) to extract milk, followed by inoculation with two strains of *Lactobacillus plantarum* DHCU 70 and *Lactiplantibacillus plantarum* MCC 5231 at 10% (*v*/*v*). This was kept for 6 h of fermentation, after which the flavoring percentage using sweetened cocoa powder was standardized to 5% for the fermented beverages.

### 2.4. Nutritional and Physicochemical Characterization of Beverage

The macronutrients and minerals of these fermented beverages were quantified using the guidelines of [17,18].

### 2.5. Physicochemical Characterization of Beverage

The beverages fermented with *L. plantarum* DHCU 70 and *L. plantarum* MCC 5231 were assessed for parameters like proximate composition, titratable acidity, pH, total soluble solids (TSS), bulk density, water absorption capacity, swell index, reducing sugar content, and total sugar content. TSS and pH were determined using a hand refractometer (HTA Instrumentation, Bangalore, India) and a pH meter (Sigma Aldrich, Bangalore, India), respectively. To determine the total titratable acidity, 10 g of each sample was mixed with 90 mL of distilled water, and the mixture was titrated with 0.1 N NaOH using 0.1% (*w*/*v*) phenolphthalein as an indicator in 95% ethanol. Color and viscosity of each beverage were assessed using a colorimeter (Konica Minolta, CM-5) and a viscometer (Brookfield, CapitaGreen, Singapore, DV-II + Pro) according to protocols of [19,20]. Reduced sugars and total sugars were quantified following the protocol of [21].

### 2.6. Fermentation Metabolites

The fermentation metabolites specifically looked at in the current study were short-chain fatty acids (SCFAs) using gas chromatography–mass spectroscopy (GC–MS), according to [22], followed by high-performance liquid chromatography (HPLC) identification of organic acids and disaccharides was achieved using [23]. Polyphenols were identified according to the HPLC setup of [24]. The beverage samples (1 g) were homogenized in 10 mL of 80% methanol containing 0.1% formic acid and sonicated for 30 min at 25 °C. After centrifugation at 10,000× *g* for 10 min, the supernatants were pooled, evaporated under reduced pressure at 40 °C, and reconstituted in 1 mL of mobile phase A (0.1% formic acid in water). Samples were filtered through 0.22 µm PTFE syringe filters prior to injection. Chromatographic separation was performed on an Agilent 1260 HPLC system equipped with a ZORBAX Eclipse Plus C18 column (4.6 × 150 mm, 5 µm). Mobile phase A was 0.1% formic acid in water, and mobile phase B was acetonitrile. The gradient program was: 0–5 min, 5% B; 5–20 min, 5–25% B; 20–30 min, 25–50% B; 30–35 min, 50–95% B; 35–38 min, 95% B; re-equilibration at 5% B for 7 min. The flow rate was 1.0 mL/min, the column temperature 30 °C, and the injection volume 10 µL. Polyphenols were detected at 280 nm and identified by comparison of retention times and UV spectra with authentic standards.

### 2.7. Microbiological Characterization of Beverage

Parameters such as cell viability, resistance to lysozyme, acid, pH, and bile salts were analyzed for each beverage sample according to [25,26].

### 2.8. In Vitro Characterization of Beverage

The beverage was extracted according to the protocol by [18]. The beverage was dissolved in an equal volume of methanol and placed in a shaker incubator (Innova 4230, New Brunswick Scientific, Edison, NJ, USA) at 37 °C for 4 h and filtered through Whatman filter paper no. 42, which was subjected to a flash evaporator to evaporate the solvent. The resultant was reconstituted with methanol (5 mg/mL). The extraction yield was calculated using initial and final weights.

#### 2.8.1. Total Polyphenol Content and Total Flavonoid Content

The total polyphenol (TPC) and total flavonoid content (TFC) were quantified using the methods described by [7,24]. Samples were also analyzed for the presence of certain polyphenols using the HPLC [24].

#### 2.8.2. Antioxidant Potential

The in vitro antioxidant properties of each fermented beverage were estimated through DPPH, FRAP, and ABTS according to [27] with slight modifications. The superoxide anion and hydroxyl radical scavenging activity of each beverage sample was determined according to the protocol suggested by [26]. Nitric oxide inhibition activity was employed to observe the anti-inflammatory potential of the beverage, which was estimated using the protocol of [28]. The activity is expressed in mg TE per 100 g of the beverage.

### 2.9. Evaluation of In Vivo Anti-Aging Potential of Beverage

The nematodes *C. elegans* (N2 wild type) were procured from the Department of Food Protection and Infestation Control, CSIR-CFTRI. They were cultured and maintained at temperatures ranging from 18 ± 2.00 °C on Nematode Growth Medium (NGM) agar plates (containing 1.2 g NaCl, 6.8 g agar, 1 g peptone, 1 M CaCl_2_, 1 M MgSO_4_, 5 mg cholesterol per liter, maintained at pH 6.5 using KPO_4_ buffer), which were seeded with OP50. Age synchronization of the nematodes was achieved following the established methods outlined by [29]. The nematodes were cultivated on agar plates for four successive generations before the commencement of the investigation. Throughout the experiments, FUdR was consistently present, with each experimental NGM plate containing approximately 40 worms taken for three independent replications. Metformin (15 µM) was used as a positive control across all in vivo analyses.

#### 2.9.1. Acute Toxicity

The toxicity tests were conducted in accordance with [30], with some modifications. The L4-stage synchronized populations were washed off twice with M9 buffer. The extracts were given in concentrations of 0.5 to 5 mg/mL to the nematodes and checked for survivability, with M9 as a negative control.

#### 2.9.2. Health Span Assays

Aging indicators like movement, pharyngeal pumping rate, and lipofuscin accumulation were observed. The movement assay was evaluated by head swing and body bending. On the 4th, 8th, and 12th day, worms were transferred to NGM. Then, the head swing times within 1 min and body bending times within 30 s of about 20 worms per group were counted under the microscope [30]. The number of pharyngeal pumps within 30 s was defined as the pharyngeal pumping rate [31]. At each assessment day, differences among treatment groups for head-swing frequency, body bend frequency, and pharyngeal pumping rate were evaluated using one-way ANOVA with Duncan’s multiple range test. Adjusted *p* values are reported, and distinct superscript letters indicate statistically significant differences within each time point. The lipofuscin fluorescence in *C. elegans* on the sixth day of the L4 stage was measured according to [32]. Worms were anesthetized using 1 M sodium azide and mounted on a 2% agarose pad. Subsequently, the fluorescence intensity was determined and imaged using fluorescence.

#### 2.9.3. Resistance to Oxidative and Thermal Stress

To assess stress resistance and ROS levels, age-synchronized *C. elegans* were cultured on NGM plates seeded with either *E. coli* OP50 or LAB until they reached day 3 of adulthood. Oxidative stress resistance was evaluated by transferring the worms to NGM plates containing 240 µM juglone, a redox cycler. Viability was scored after 4 h of continuous exposure. Thermal stress sensitivity was determined by subjecting the worms to a 3 h heat shock at 35 °C, followed by a 2 h recovery period at 20 °C. Survival was assessed for 40 worms per an NGM plate, with three independent readings, using the touch provocation method.

Intracellular ROS levels were measured using the fluorescent probe H_2_DCF-DA. At specific time points, worms were collected, washed, and incubated with 50 µM H_2_DCF-DA for 30 min at 20 °C in the dark. Following incubation, fluorescence microscopy was used to measure DCF fluorescence signals, which were quantified using ImageJ software version 2023 (ImageJ 1.54d).

#### 2.9.4. Production of Reactive Oxygen Species

The ROS production in the *C. elegans* was assessed by following the methodology described by [33].

#### 2.9.5. Lifespan Assay

Lifespan analysis was conducted using approximately 40 synchronized L1 larvae per well of a 24-well plate. The larvae were cultured in S-complete media (S-basal media, 1 M potassium citrate, and trace metals solution) containing *E. coli* OP50 (60 μL per mL of media, 1.2 × 10^9^ CFU/mL) and an antibiotic solution (carbenicillin at 50 μg/mL and amphotericin B at 0.1 μg/mL). FUdR (0.06 mM) was added to each well, followed by 1–15 μM of beverage extracts at 48 and 72 h post-seeding. Treatment began on day 0, and assessments were conducted every other day. During each assessment, *C. elegans* were examined at 40X magnification for signs of life (e.g., pharyngeal movement) and returned to an incubator at 18 °C at 4 day intervals for 28 days, with 120 worms per reading (3 plates of 40 individual-NGM plates), followed by three independent readings. Death was defined as the absence of movement or response to microscopic light. Animals that crawled off the plate, experienced rupture or bagging, or became contaminated were excluded from analysis [29].

#### 2.9.6. Biochemical Parameters

Antioxidant enzymes like superoxide dismutase (SOD), malondialdehyde (MDA), glutathione (GSH), and catalase (CAT) were analyzed according to the protocols [34]. Preparation of samples (sample size *n* = 300 ± 25.00) with three biological triplicates was performed according to [35].

### 2.10. Sensory Evaluation

The 9-point hedonic scale was used to assess the sensory attributes of the fermented beverages and the control. Informed consent was obtained from participants, who were provided with specific information indicating that the beverage is composed of regularly consumed ingredients and a probiotic strain identified as GRAS (Supplementary Materials).

### 2.11. Statistical Analysis

Each measurement was conducted three times. The data was analyzed using one-way analysis of variance (ANOVA), and differences between means were compared using Duncan’s multiple range test (DMRT), which was performed using GraphPad Prism version 5 (San Diego, CA, USA). A significance level of *p* ≤ 0.05 (95% CI) was used to identify significant differences between the mean values. For in vivo studies, biological replicates consisted of independent worm populations (approximately 40 worms per replicate) initiated from separate, synchronized egg stages and maintained on individual NGM plates. Each treatment group included three biological replicates (total *n* ≈ 120 worms per group). Biochemical assays were performed on pooled worm homogenates (total *n* ≈ 300 worms per group) from each biological replicate, with technical measurements conducted in triplicate. Data normality and variance homogeneity were verified prior to parametric testing using Shapiro–Wilk and Levene’s tests, respectively. Daily survival scoring in lifespan analyses was performed using standard viability criteria. Kaplan–Meier survival curves were compared using log-rank tests, with both mean and median lifespan values reported alongside 95% confidence intervals. Gompertz mortality modeling was performed to estimate aging rate parameters and initial mortality with corresponding standard errors and goodness-of-fit statistics) [36]. Behavioral and stress resistance assays were analyzed using one-way ANOVA with Duncan’s multiple range test for post hoc comparisons. Respective statistical results include F-statistics with degrees of freedom, exact *p*-values, and effect sizes (η^2^). All statistical analyses applied a significance threshold of *p* ≤ 0.05. Biological replicates (*n* = 3 independent worm populations) were used exclusively for the *C. elegans* in vivo studies, whereas beverage analyses were performed using technical triplicates.

## 3. Results and Discussion

### 3.1. Characterization of Probiotic Beverage

#### 3.1.1. Nutritional Composition

**Figure 1:**
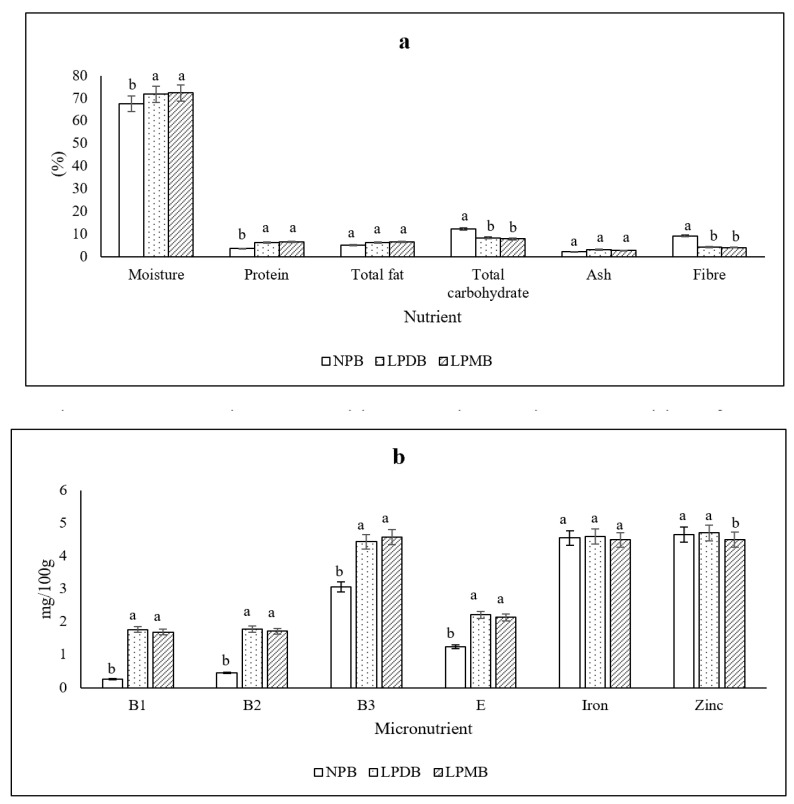
(**a**) Macronutrient composition (**b**) Micronutrient composition of NPB (Non-probiotic beverage); LPDB (*L. plantarum* DHCU70 probiotic beverage); LPMB (*L. plantarum* MCC 5231 probiotic beverage). All of the values are mean ± standard error of the mean (SEM). Different superscript letters indicate significant differences within the same macro- or micronutrient category as determined by one-way ANOVA followed by Duncan’s multiple range test (DMRT, *p* < 0.05).

The beverages fermented with *L. plantarum* DHCU 70 (LPDB) (71.82 ± 0.41%) and *L. plantarum* MCC 5231 (72.30 ± 0.76%) (LPMB) showed significantly (*p* < 0.05) increased moisture content than its control (67.57 ± 0.59%), while there was a significant (*p* < 0.05) increase in its protein from 3.58 ± 0.84% in the control to 6.13 ± 1.00% and 6.42 ± 0.33% in the beverages fermented with *L. plantarum* DHCU 70 and *L. plantarum*, respectively *(*Figure 1a). The total fat of the beverages fermented with *L. plantarum* DHCU 70 and *L. plantarum* MCC 5231 was 6.35 ± 0.19% and 6.51 ± 0.18% compared to the control (5.17 ± 0.17%). We observed a significant decrease in the total carbohydrate and fiber contents post-fermentation with a mean difference of 6.34% and 4.95%, respectively, compared to the unfermented control. However, the total ash content and the contents of minerals like calcium, potassium, magnesium, phosphorus, iron, and zinc showed no significant difference between the control and the pearl millet beverages fermented with *L. plantarum* DHCU 70 and *L. plantarum* MCC 5231. Also, a critical observation of the increased contents of B and E vitamins post-fermentation was observed. The vitamin B_1_, B_2_, B_3_ increased from 0.25 ± 0.05, 0.45 ± 0.14, 3.06 ± 0.10 mg 100 g^−1^ in the control to 1.76 ± 0.05, 1.77 ± 0.06, 4.43 ± 0.38 mg 100 g^−1^ in the beverage fermented with *L. plantarum* DHCU 70 while similar increase in B1 (1.68 ± 0.03 mg 100 g^−1^), B2 (1.71 ± 0.07 mg 100 g^−1^) and B3 (4.57 ± 0.43 mg 100 g^−1^) was found in the beverage fermented with the strain *L. plantarum* MCC 5231, respectively (Figure 1b).

Literature shows fermentation to recompose the proximate composition of plant-based food. Studies showed a 12.5% increase in the protein content of pearl millet during fermentation. Literature shows that fermentation decreases the original carbohydrate content as in composite complementary food products made of sorghum–amaranth, millet–pumpkin, sorghum–pumpkin–amaranth, and millet–pumpkin–amaranth [37]. Also, fermentation using *Lactobacillus plantarum* showed reduced total carbohydrate content in cereal and pseudo-cereal flours [38]. Fermentation decreases the content of dietary fiber due to the enzymatic breakdown of the fiber structure by lactic acid bacteria [39]. During the fermentation of the coco-yam flour, co-fermented millet, and cowpea mixture and, the fiber contents decreased throughout fermentation. In the same way, ref. [40] reported that the fiber content of composite flour made of breadfruit and cowpea decreased during fermentation.

#### 3.1.2. Physicochemical Characteristics

The physicochemical characteristics influence the composition, sensory quality, rheological and chemical behavior, stability, and shelf life of food products, specifically beverages, in which each of the analyzed parameters indexes the product’s appearance, flavor, taste, rheology, and physical stability. Post fermentation physicochemical changes in the study (Table 1) include significant increase in the titratable acidity from 2.36 ± 0.17 g/L in control to 6.23 ± 0.08 and 6.49 ± 0.29 g/L in fermented beverages (LPDB and LPMB) with simultaneous decrease in pH from 7.53 ± 0.07 in control to 5.17 ± 0.67 in the beverage fermented with *L. plantarum* DHCU 70 and 4.15 ± 0.18 in the beverage fermented with *L. plantarum* MCC 5231, respectively. The fermented beverages showed less TSS with 10.09 ± 0.31 °B in the beverage fermented with *L. plantarum* DHCU 70 and 09.8 ± 0.28 °B in the beverage fermented with *L. plantarum* MCC 5231 compared to the control (14.83 ± 0.25 °B). The bulk density of the fermented beverages with *L. plantarum* DHCU 70 (0.86 ± 0.01 g/cm^3^) and *L. plantarum* MCC 5231 (0.84 ± 0.03 g/cm^3^), while in the control, it was 0.58 ± 0.13 g/cm^3^, respectively. The probiotic beverages made from fermented millet (LPDB and LPMB) showed relatively lower lightness values (L* = 30.58 ± 0.02 and 32.45 ± 0.02), giving them a darker visual tone. The a* and b* values were moderate (positive), suggesting a reddish-yellow coloration. When comparing overall color variation (ΔE), LPMB (68.45 ± 0.04) showed slightly brighter tones with less deviation in color compared to NPB (70.74 ± 0.07) and LPDB (70.07 ± 0.03). In terms of texture, the viscosity levels varied significantly. NPB showed higher consistency at 856.00 ± 22.06 cP, while LPDB and LPMB were lower (Table 1). This reduction in thickness correlates with the decrease in total soluble solids (TSS) post-fermentation. The study observed a significant reduction in sugar content, with reducing sugar content decreasing from 7.59 ± 0.14 mg 100 mL^−1^ WB in the control to 3.20 ± 0.14 mg 100 mL^−1^ WB and 3.18 ± 0.03 mg 100 mL^−1^ WB in the fermented beverages LPDB and LPMB, while the total sugar content decreased from 12.24 ± 0.72 to 4.78 ± 0.19 mg 100 mL^−1^ WB in the beverage fermented with, while the total sugar content decreased to 4.44 ± 0.21 mg 100 mL^−1^ WB in the beverage fermented with a mean decrease of 7.63 mg 100 mL^−1^ respectively. The average decrease in total sugar was 62% which correlates with an average decrease in total carbohydrate (34%) and fiber contents (36%), post-fermentation.

### 3.2. Post-Fermentation Metabolites

The probiotic beverages showed post-fermentation metabolites (Table 2) including polyphenols such as gallic acid, epigallocatechin gallic acid, di-hydroferulic acid, quercetin, caffeic acid, 4-vinyl guaiacol, which were not in NPB, which showed vanillic acid, catechin, kaemferol, and rutin (Figure S1). Gas chromatography mass spectrometry (GC MS) revealed the presence of short-chain fatty acids (SCFAs), including valerate, acetate, iso-butyrate, propionate, iso-propionate, and methyl-propionate post-fermentation (Figure S2); however, no SCFAs were identified in the control. The probiotic beverages showed organic acids such as citric acid, formic acid, lactic acid, and malic acid, while NPB showed the presence of oxalic acid (Figure S3). Free saccharides like mannose, xylitol, and fructose were identified in LPDb and LPMB, while NPB showed the presence of glucose (Figure S4).

### 3.3. Microbiological Parameters

The viability of the probiotic strain (Table 3) post 12 h fermentation was 8.8 ± 0.07 and 9.2 ± 0.04 log CFU/mL in beverages fermented with *L. plantarum* DHCU 70 and *L. plantarum* MCC 5231, respectively. The strain *L. plantarum* DHCU 70 showed higher resistance to decreased pH and bile salts, with effective 8.5 ± 0.15 and 8.6 ± 0.11 log CFU/mL, showing its sustenance through the stomach and duodenum, while the strain *L. plantarum* MCC 5231 showed 8.1 ± 0.17 and 8.4 ± 0.10 log CFU/mL. Lysozyme, an antimicrobial enzyme present in small intestinal juice, simulates its harsh conditions, and both probiotic strains in the study showed their viability, maintaining their effective counts of 8.3 ± 0.24 and 8.7 ± 0.009 log CFU/mL in *L. plantarum* DHCU 70 and in *L. plantarum* MCC 5231. The study shows that the strains survive harsh gut conditions to reach the large intestine for colonization.

### 3.4. In Vitro Studies

#### 3.4.1. Yield Efficiency

The study showed an extraction yield rate (%) of 40 ± 2.00%, 42 ± 2.51% and 40 ± 1.52% in control and pearl millet beverages with probiotic strains of *L. plantarum* DHCU 70 and *L. plantarum* MCC 5231, respectively.

#### 3.4.2. Total Polyphenol and Total Flavonoid Content

**Figure 2:**
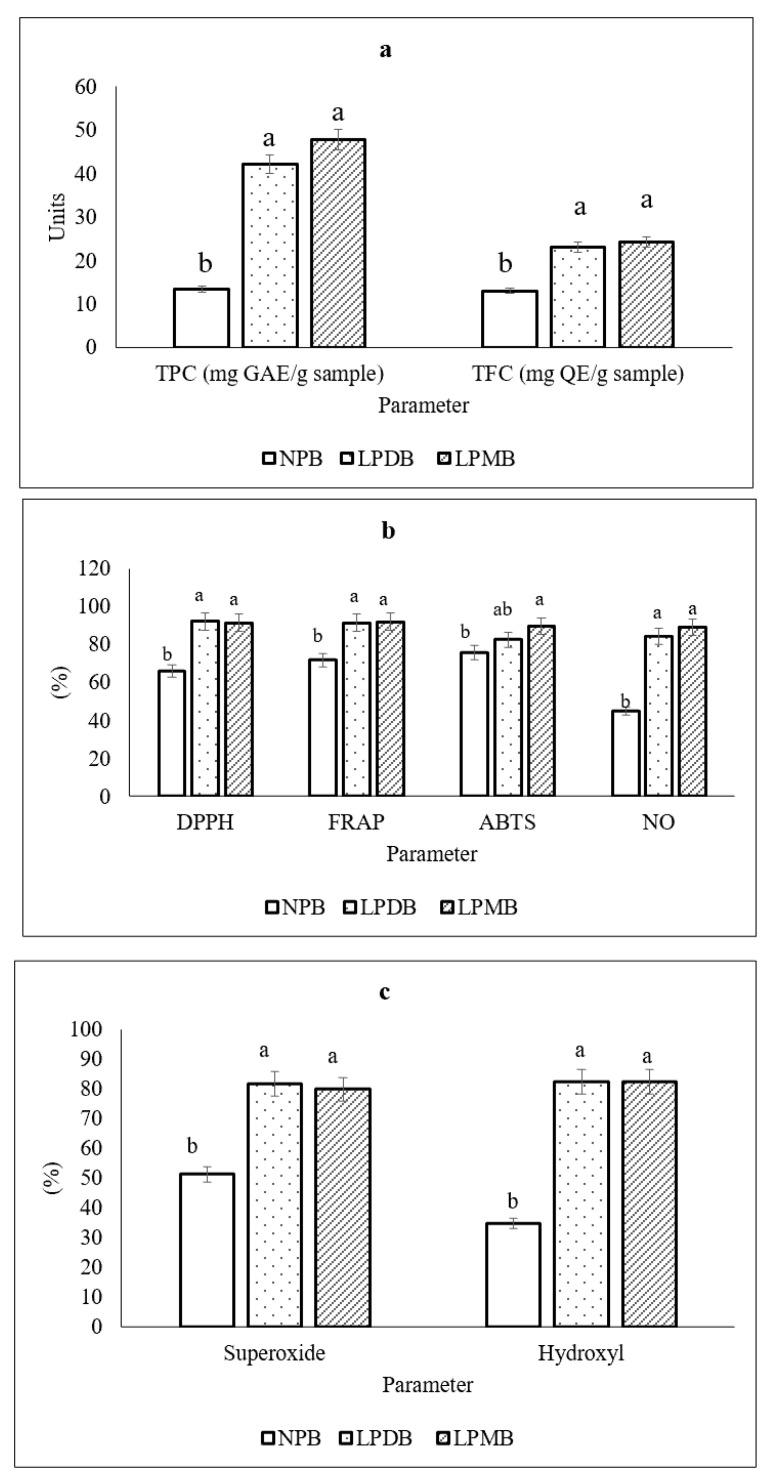
(**a**) Total polyphenolic and flavonoid content. (**b**) Radical scavenging and anti-inflammatory potential. (**c**) ROS scavenging potential of **NPB** (Non-probiotic beverage); **LPDB** (*L. plantarum* DHCU70 probiotic beverage); **LPMB** (*L. plantarum* MCC 5231 probiotic beverage). All of the values are mean ± standard error of the mean (SEM). Mean values with the same superscript letters are not significantly different, whereas those with the different superscript letters are significantly (*p* < 0.05) different, as judged by Duncan’s multiple range test.

There was a significant increase in TPC and TFC (Figure 2a) post-fermentation, amounting to 42.10 ± 2.65 mg GAE/100 g and 23.12 ± 2.73 mg QE/100 g in the fermented beverage with the strain *L. plantarum* DHCU 70, while the values of TPC and TFC were 47.76 ± 1.37 mg GAE/100 g and 24.21 ± 0.98 mg QE/100 g, respectively, in the fermented beverage containing the strain *L. plantarum* MCC 5231. The TPC and TFC values of the control were observed to be 13.38 ± 0.40 mg GAE/100 g and 13.01 ± 1.18 mg QE/100 g, respectively. A similar increase in TPC was seen in a study conducted by [41], where biofortified pearl millet fermented by *Lactobacillus rhamnosus* GG 347 for 16 h showed 39.68 mg GAE/100 g, while the TFC was reported to be 20.70 ± 0.57 mg QE/100 g. Another study reported that fermentation of ganmai dazao decoction by lactic acid bacteria showed increased polyphenol content but a decrease in TFC from 1.70 ± 0.04 mg/mL to 0.82 ± 0.04 mg/mL at 0–10 h and then increased at 10–14 h, which was 1.10 ± 0.03 mg/mL reported [42]. The enhancement of phenolic content can be attributed to the ability of bacteria to produce β-glycosidase, an enzyme that facilitates the hydrolysis of β-glycosides into isoflavone aglycones [43]. Additionally, studies highlight the capability of *L. plantarum* to generate esterases that break down ester bonds in glycosides, thereby releasing soluble conjugated or insoluble bound phenolic compounds from plant cell walls [44]. The basis for the increase in flavonoid content was explained to be the ability of LAB to form compounds as metabolic byproducts that enhance the concentration of accessible bioactive molecules [45]. The results are most likely related to the LAB’s metabolic processes and may result in the breakdown of highly complex phenolic substances or the release of phenolic substances (such as flavonoids), which are linked to the structure of food materials [46].

#### 3.4.3. In Vitro Antioxidant and Anti-Inflammatory Potential

Our previous study showed that pearl millet has the potential for antioxidation by 60.84% [18], while fermentation enhanced DPPH radical scavenging by 37% in both fermented beverages. Ferrous chelation potential increased from 71.69 ± 0.09 to 91.45 ± 0.006 mg TE 100 g^−1^ in the LPDB beverage, while the FRAP value amounted to 91.83 ± 0.03 mg TE 100 g^−1^ in the fermented beverage LPMB. Fermentation with *L. plantarum* showed ABTS scavenging potential of 82.51 ± 0.04 mg TE 100 g^−1^ in LPDB (Figure 2b). and 89.74 ± 0.04 mg TE 100 g^−1^ in the LPMB. The study also observed the ROS scavenging ability, which showed superoxide % inhibition increase from 51.40 ± 0.98% in unfermented beverage control to 81.77 ± 0.03% and 79.92 ± 0.02% in fermented beverages, LPDB and LPMB, while they showed % inhibition of hydroxyl radicals up to 82.58 ± 0.02% and 82.33 ± 0.04%, respectively (Figure 2c). The average nitric oxide (NO) inhibition was shown to be 85.74 ± 5.26% and the fermentation beverages each showed an enhancement with an average NO inhibition of 86.68 ± 0.01%. A similar study showed an increasing trend in the DPPH free radical scavenging from 15.15 ± 1.17% to 92.14 ± 0.20% which was 6.08 times higher at the end of fermentation [42]. Also, the same reported hydroxyl free radical clearance was 36.49% higher from 57.25 ± 2.61% to 78.14 ± 0.62%, after fermentation. The increase in DPPH suggests that lactic acid fermentation could increase the compounds with characteristics of proton-donating availability [47]. They act as reducing agents, free radical scavengers, and singlet oxygen quenchers. Phenolic compounds have significant antioxidant activities, which are mainly attributed to the hydrogen atom transfer or electron donation to free radicals) [48]. After fermentation, the scavenging rate of DPPH and hydroxyl free radicals increases significantly, and the enhancement of antioxidant activity appears to be dependent on an increase in phenols [42].

### 3.5. In Vivo Studies

*C. elegans* were treated with beverages (NPB, LPDB, and LPMB) and control (M9 buffer) after which various oxidative stress and aging parameters were analyzed.

#### 3.5.1. Effect of Probiotic Beverage Extracts on Acute Toxicity on *C. Elegans*

The acute toxicity of LPDB and LPMB beverages is shown by the viability of worms, wherein a concentration range between 0.5 and 3 mg/mL was found to be non-toxic after 24 h when compared to the control group (Table S1).

#### 3.5.2. Health Spans Assays

##### Movement Assay

**Figure 3:**
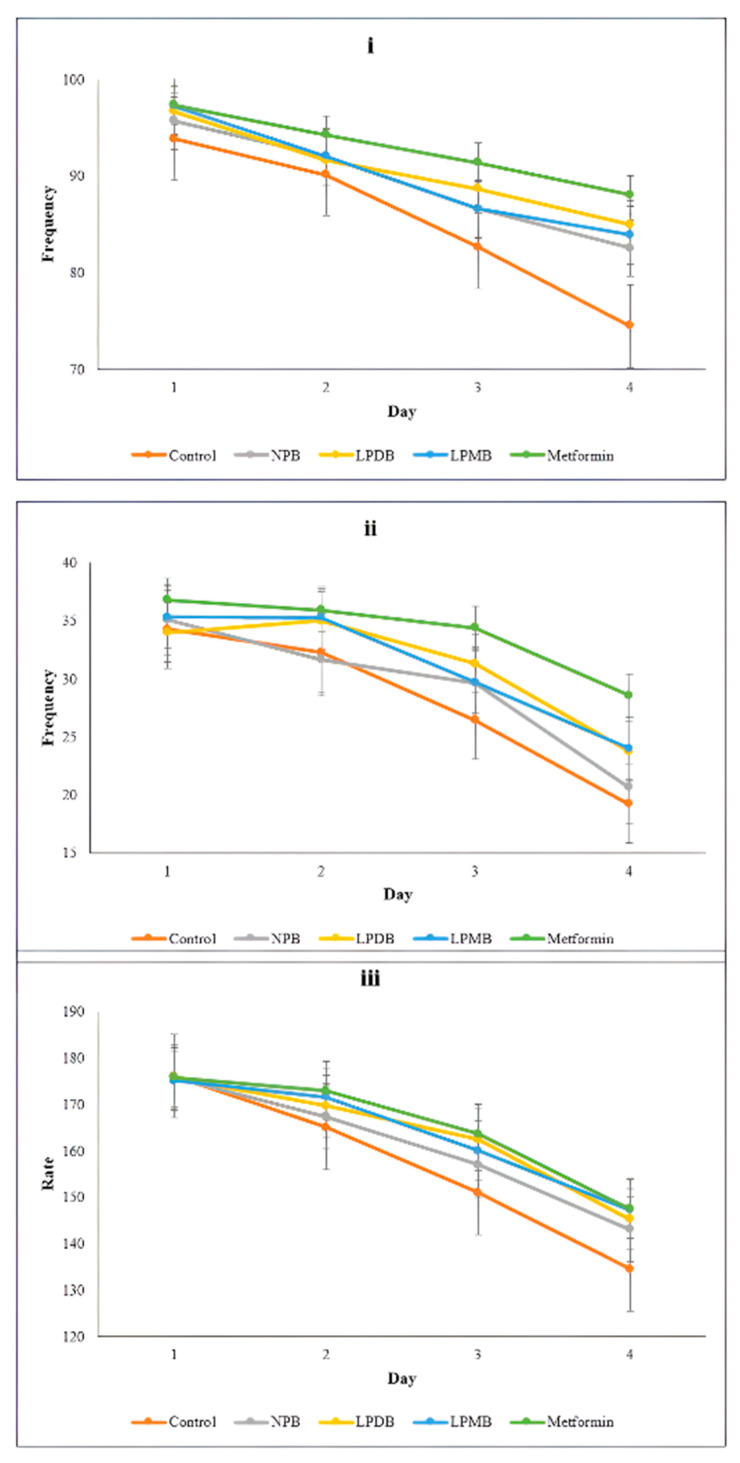
Effect of treatments on (**i**) head-swing frequency, (**ii**) body bend frequency, and (**iii**) pharyngeal pumping rate. Control (M9 buffer); NPB (Non-probiotic beverage); LPDB (*L. plantarum* DHCU70 probiotic beverage); LPMB (*L. plantarum* MCC 5231 probiotic beverage). Statistical significance for within-day comparisons across all treatments was determined using one-way ANOVA followed by Duncan’s multiple range test to adjust for multiple comparisons.

Aging in *C. elegans* shows physiological and biochemical characteristics similar to those in humans. The model was then used to determine the effects of gerobiotic beverages on aging indicators. Among those indicators, the movement and pharyngeal pumping reflect the aging process in nematodes and are considered key physiological indicators to evaluate aging in *C. elegans*. Therefore, in the study, the head-swing frequency was higher in probiotic beverage-treated worms (84.95 ± 2.56 and 83.90 ± 2.77) compared to NPB (82.55 ± 4.47) and control (74.50 ± 1.82) on the 12th day, while the positive control group showed the highest (88.05 ± 2.56). The study observed an average body-bend frequency of 34.65 ± 0.62 initially across all groups (Figure 3). However, the decrease was subtle in LPMB, with a mean difference from initial to final day frequency of 11.30 and significantly higher (24.00 head swings per minute), followed by LPMB (23.75 ± 2.76), NPB (20.65 ± 2.66), with the least frequency observed in the control (19.20 ± 2.87). The worms treated with the fermented probiotic beverage showed higher pharyngeal pumping rate by 7.49% and 8.82% than the control, while worms treated with NPB had a 6.35% higher pharyngeal pumping rate per minute. A similar study by [16] showed better performance in each indicator (sinusoidal frequency 15.2% higher, pharyngeal frequency by 12.1% on the 8th day) with treatment by millet bran peptides.

#### 3.5.3. Lifespan Extension Assay

**Figure 4:**
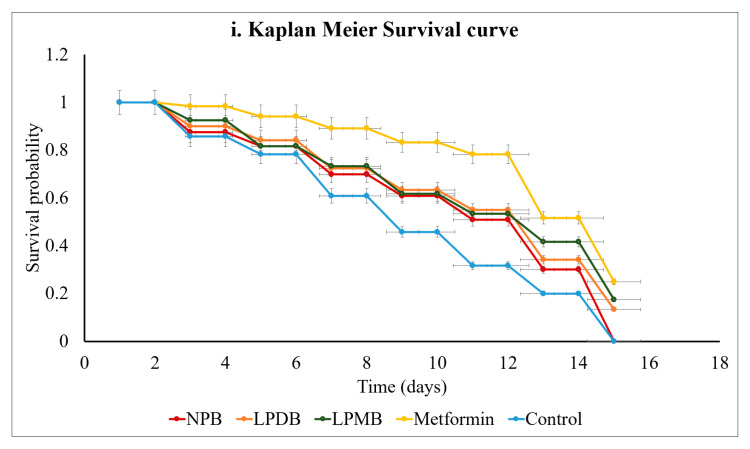

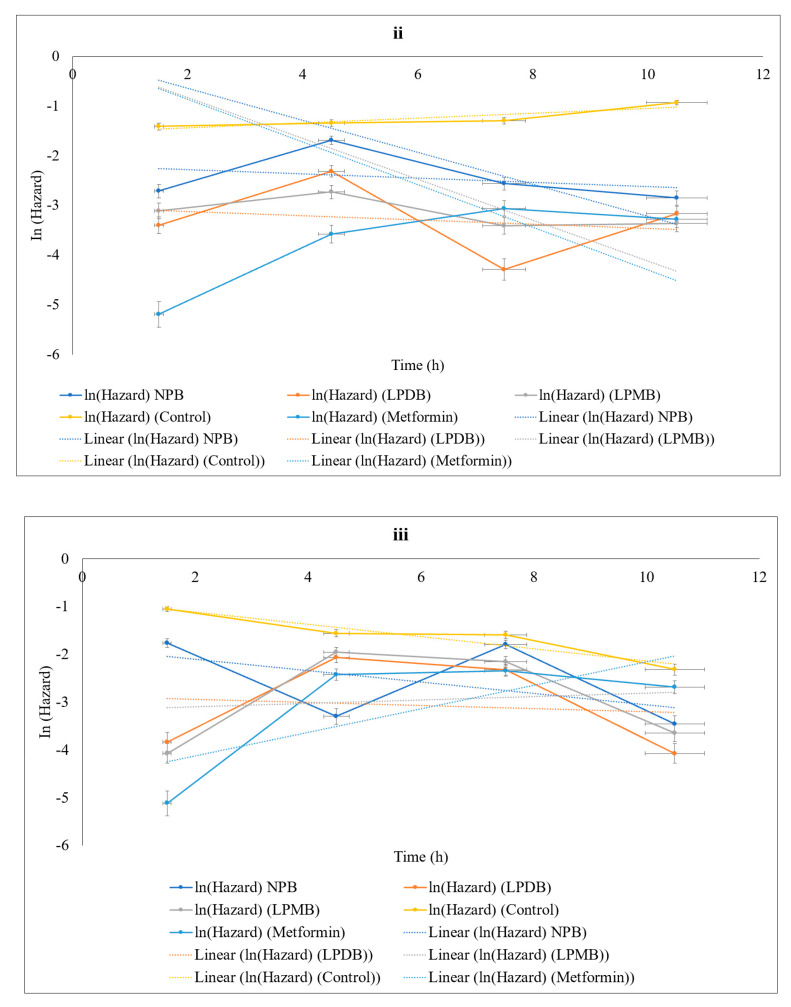
Effect of treatments on (**i**) lifespan using Kaplan–Meier Survival curve, (**ii**) thermal stress tolerance, and (**iii**) H_2_O_2_-induced oxidative stress tolerance. Control (M9 buffer); NPB (Non-probiotic beverage); LPDB (*L. plantarum* DHCU70 probiotic beverage); LPMB (*L. plantarum* MCC 5231 probiotic beverage); Metformin (positive control).

In this study, we found that the beverage with abundant polyphenols, short-chain fatty acids, organic acids, and free sugars was observed to extend the lifespan of *C. elegans* (Figure 4i). Survival analysis using the Kaplan–Meier method was conducted for nematodes treated with the Control (M9 buffer), NPB, LPDB, LPMB, and Metformin (Positive control), over a 28-day period, with all groups starting at 100% survival at day 0. Divergence in survival rates at initial time points revealed distinct efficacies of the treatments.

From day 0 to day 4, survival probabilities spanned from 85.83% in the Control to 98.33% in Metformin-treated nematodes. Notably, LPDB (90%) and LPMB (92.5%) exceeded both Control and NPB (87.5%) survival rates, establishing their superior efficacy early. With the study progressing beyond day 16, the Control survival sharply decreased to 20% by day 24 and 0% by day 28, while LPDB and LPMB substantially preserved survival, maintaining 34.17% and 41.67%, respectively, at day 24, which surpassed both Control and NPB (30%), though remained slightly below Metformin (51.67%). At day 28, when the Control and NPB survival reached zero, *C. elegans* treated with LPDB (13.33%) and LPMB (17.5%) continued to exhibit survival, though on par or slightly lower than Metformin (25%). These findings highlight that LPMB and LPDB significantly extended the survivability of the Control and NPB, while performing comparably to, though somewhat less effectively than, Metformin, the positive control. NPB provided a moderate survival benefit but was consistently surpassed by the LPDB and LPMB interventions. These results support the potential of LPMB and LPDB as effective interventions to mitigate stress and aging-related mortality. Statistical comparison using log-rank tests confirmed the significance of the differences between groups (*p* < 0.05).

#### 3.5.4. Tolerance to Stress Conditions

*C. elegans* subjected to the interventions in the study were analyzed for the time-course of the natural logarithm of mortality hazard (In(Hazard)) under thermal and oxidative stress, along the time midpoints of 1.5, 4.5, 7.5, and 10.5 h (Figure 4ii,iii). From initial to final time points, the control showed a high hazard (In(Hazard)) of −1.41, while NPB (−2.71), LPDB (−3.4), and LPMB (−3.11) showed lower hazards. Metformin-treated nematodes showed reduced hazard ((In(Hazard)) of −5.19, all of which, at the end of the study, showed (In(Hazard)) of LPDB (−3.17), LPMB (−3.36), Metformin (−3.28), and NPB (−2.85). However, across time points, control showed an increasing trend was seen in control with (In(Hazard)) of −0.93. This implies the protective effects of the treatments against control, while also showing that fermented beverages—treated nematodes showed a reduction in mortality risk compared to that of control across the time points of the study (Figure 4ii,iii). A similar pattern was observed under oxidative stress, where, at 1.5 h, Metformin showed the lowest risk (ln(Hazard) = −5.115), with LPDB and LPMB also showing reduced hazards (−3.828, −4.069) compared to the Control (−1.048) and NPB (−1.761). With progression over time, the natural logarithm of hazard increased (ln(Hazard) values became less negative) across all groups, but Metformin and the two LP groups consistently maintained lower hazard levels compared to Control and NPB. For more clarity, at 4.5 h, Metformin hazard was −2.423, LPDB was −2.064, LPMB was −1.948, while the Control and NPB were −1.555 and −3.297, respectively. The pattern persisted through later time points (7.5 and 10.5 h), with Metformin and the LP groups always showing lower mortality hazards compared to the Control, and NPB showed intermediate mortality risk (In(Hazard)). Gompertz regression of instantaneous mortality hazard revealed that LPDB and LPMB reduced the rate-of-aging parameter (b*b*), indicating a suppression of stress-induced mortality acceleration. Specifically, the slope of log mortality versus time was lower in treated groups, consistent with a delayed onset or systemic mitigation of age-related physiological decline under acute stress. Non-fermented controls showed intermediate resistance, while negative controls exhibited both rapid early mortality and higher Gompertz b*b* values, showing their increased vulnerability.

#### 3.5.5. Lipofuscin Accumulation Assay

**Figure 5:**
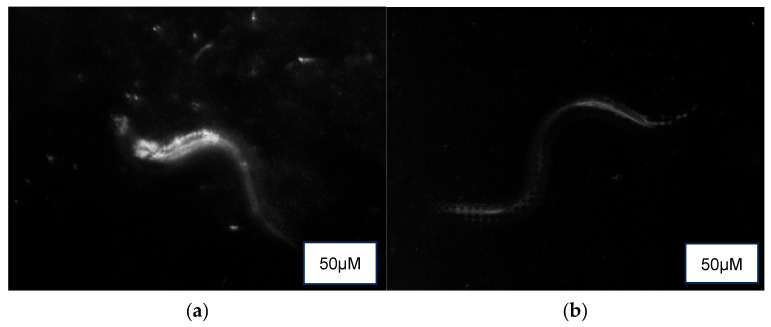

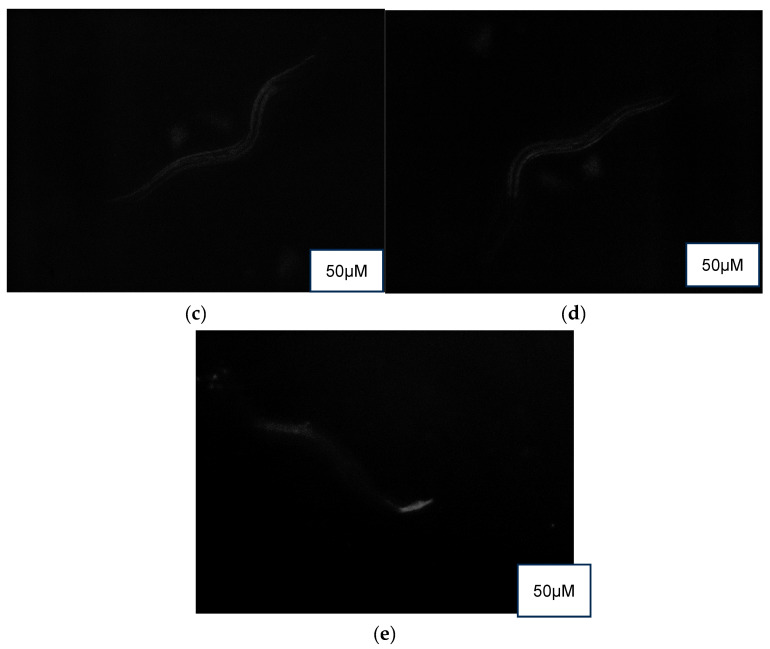
Auto-fluorescence detection of lipofuscin in (**a**) Control (M9 buffer), (**b**) NPB (non-probiotic beverage), (**c**) LPDB (*L. plantarum* DHCU70 probiotic beverage), (**d**) LPMB (*L. plantarum* MCC 5231 probiotic beverage), (**e**) Metformin (positive control).

Auto-fluorescence (Figure 5) revealed a higher presence of lipofuscin in the control (M9 buffer) compared to the treatment groups of NPB, LPDB, and LPMB.

#### 3.5.6. Reactive Oxygen Species Detection Assay

**Figure 6:**
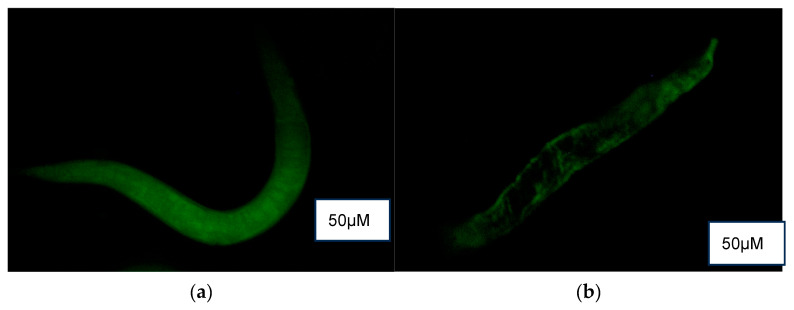

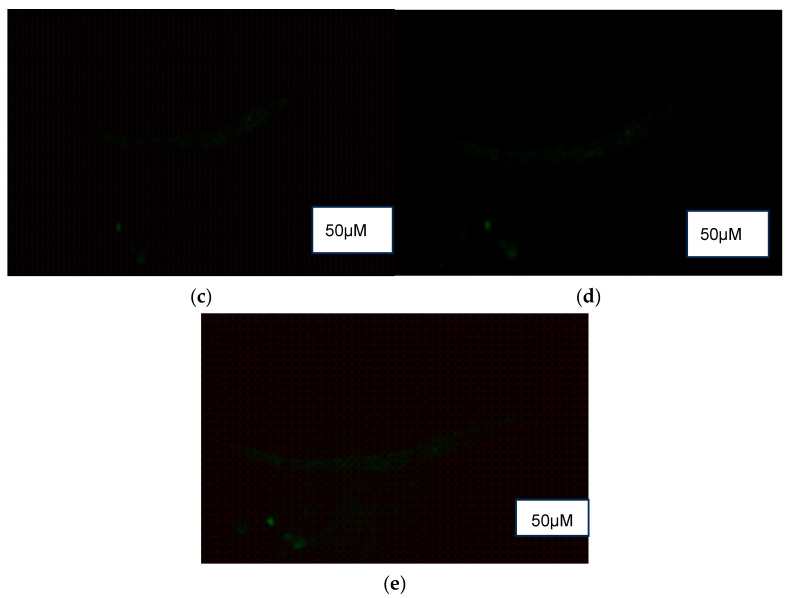
ROS detection using DCF fluorescence in (**a**) Control (M9 buffer), (**b**) NPB (non-probiotic beverage), (**c**) LPDB (*L. plantarum* DHCU70 probiotic beverage), (**d**) LPMB (*L. plantarum* MCC 5231 probiotic beverage), (**e**) Metformin (positive control).

DCF fluorescence (Figure 6) revealed a higher presence of reactive oxygen species (ROS) in the control (M9 buffer) compared to treatment groups of NPB, LPDB, and LPMB.

#### 3.5.7. Biochemical Parameters

Gerobiotic supplementation showed improved antioxidant and oxidative stress markers in *C. elegans* (Table 4). The control group showed 23.49 ± 3.10% CAT inhibition, 0.52 ± 0.13 U/mg protein SOD activity, 12,620 ± 17.11 nmol/mg protein GSH, and 0.89 ± 0.04 nmol/mg protein MDA. Worms treated with NPB showed reduced CAT inhibition to 11.37 ± 4.32%, increased SOD (0.83 U/mg protein) and GSH (13,952 ± 21.14 nmol/mg protein) with lowered MDA (0.76 nmol/mg protein). Treatment with probiotics LPDB and LPMB decreased CAT inhibition to 8.11 ± 2.52% and 8.67 ± 1.95%, increased SOD activity to 1.53 ± 0.24 and 1.38 ± 0.44 U/mg protein, and GSH levels to 16,875 ± 26.91 and 15,067 ± 23.63 nmol/mg protein. Both LPDB and LPMB-treated nematodes showed reduced MDA to 0.65 ± 0.03 and 0.61 ± 0.05 nmol/mg protein, respectively, performing closer to Metformin (positive control), which showed the highest with 7.65 ± 1.71% CAT inhibition, 1.64 ± 0.23 U/mg protein SOD activity, 15,383 ± 22.41 nmol/mg protein GSH and 0.49 ± 0.07 nmol/mg protein MDA. A similar study by Kumaree et al. (2023) [49] showed that feeding *C. elegans* with *Lactobacillus paracasei* HII01 significantly increased their lifespan and reduced aging markers, such as lipofuscin accumulation, with enhanced antioxidant defense enzymes, improving oxidative stress resistance.

### 3.6. Sensory Evaluation of Probiotic Beverage

The fermented and control beverages were evaluated for sensory attributes including appearance, color, flavor, taste, consistency and overall acceptability in which the study revealed an increase in panel preference by 8% in beverage fermented with *L. plantarum* DHCU 70 and by 16% in *L. plantarum* MCC 5231 with overall acceptability scores of 8.15 ± 0.83 and 8.79 ± 0.30 out of 9, respectively.

## 4. Discussion

Fermentation has been a vital food processing technique for thousands of years, valued for its ability to enhance shelf life, nutritional content, and introduce beneficial probiotics. Unlike fermentation, ultra-processing also prolongs shelf life, but is associated with negative health impacts (Lane et al., 2024) [50]. The study was conducted to investigate the post-fermentation changes in the physicochemical, nutritional, bioactive phenolic content, and sensory profile of a pearl millet-based probiotic beverage. Nutritional analysis revealed a significant (*p* < 0.05) increase in moisture content, protein, vitamins B and E, while a decrease in total carbohydrate and dietary fiber post 6 h fermentation, similar to the findings of Srivastava et al. (2024) [51]. Studies have shown that probiotic fermentation could affect color parameters (L*, a*, b*) and overall appearance of beverages through compositional and metabolic changes, leading to products with different brightness and hue compared to controls. Additionally, viscosity is often reduced in probiotic-enriched formulations; this has been linked to enzymatic activity from probiotics, which can break down thickening agents or matrix structure, resulting in a thinner beverage consistency compared to non-probiotic samples [52]. This fermentation-dependent increase in nutritional value may be due to breaking down anti-nutritional factors like phytates through microbial enzymes, which enhances mineral bioavailability, protein digestibility, and enriches vitamins and bioactive compounds [53,54]. The bioactive profile in terms of total polyphenolic and flavonoid content was enhanced with polyphenols such as catechin, vanillic acid, kaempherol, and epigallocatechin gallic acid post-fermentation. The metabolic activity of L. plantarum was studied to generate bioactive peptides and short-chain fatty acids during fermentation, as observed in the current study, which may play roles in antioxidant defense and modulation of inflammation [55,56]. The study highlights a significant increase in antioxidant and anti-inflammatory potential across all assays, including DPPH radical scavenging (37% increase), FRAP (27% increase), and ABTS scavenging potential (20%), respectively. The fermented beverages demonstrated substantial superoxide radical inhibition (81.77% in LPDB and 79.92% in LPMB), compared to 51.40% in the control. Hydroxyl radical inhibition also reached over 82% in both fermented treatments. Additionally, nitric oxide inhibition showed minor improvement, increasing from 85.74% in the control to an average of 86.68% in fermented samples. Substantial studies show that probiotic fermentation enhanced the in vitro antioxidant, anti-inflammatory, and free radical scavenging potential associated with increased total phenolic content [57,58]. In vivo studies using *C elegans* showed significant differences among the treatment groups in physiological indices like head-swings, body bend, and pharyngeal pumping rate, ability to tolerate thermal and oxidative stress, while fluorescence showed lower accumulation of lipofuscin and ROS in treatment groups. The study also highlights the fermentation-dependent enhancement of longevity, with LPMB showing superior efficacy. This suggests that bioactive compounds in these mixtures modulate conserved aging pathways or enhance stress resistance mechanisms in *C. elegans*. Qin et al. (2022), Govindhan et al. (2023), and Li et al. (2023) [16,26,30] showed a similar increase in physiological parameters, stress tolerance, and median lifespan when *C. elegans* were treated with plant polyphenol extracts, barnyard-based gerobiotic beverage, and millet bran peptides. Xu et al. (2023) [59] found that probiotic fermented ginseng increased antioxidant enzymes (T-SOD, GSH-PX, and CAT) while decreasing MDA and ROS levels in *C. elegans*, resulting in an extended lifespan and increased stress resistance via SKN-1 and SOD-3 pathway activation. Furthermore, Hu et al. (2022) [60] discovered that *Pediococcus acidilactici* from fermented pickles increased longevity in *C. elegans* by reducing ROS accumulation and enhancing antioxidant defense mechanisms, as well as decreasing lipid accumulation and inflammatory responses by upregulating sod-3 expression. The observed longevity effects in *C. elegans* likely reflect the synergistic interaction between these established probiotic strains and the pearl millet bioactive matrix. The fermentation process enabled both strains to metabolize millet phenolic precursors, generating bioactive metabolites, including gallic acid, epigallocatechin gallate, and various short-chain fatty acids that were absent in the unfermented control.

## 5. Conclusions

The study was carried out to observe post-fermentation changes in the physicochemical, nutritional, bioactive phenolic content, and sensory profile of pearl millet probiotic beverage. Also, the probiotic fermentation enhanced the in vitro antioxidant, anti-inflammatory, and free radical scavenging potential of fermented beverages (LPDB, LPMB). Nutritional analysis revealed a significant (*p* < 0.05) increase in moisture content, protein, vitamins B and E, while a decrease in total carbohydrate and dietary fiber post 6 h fermentation. Physicochemical characteristics were observed to be affected by fermentation, with an increase in titratable acidity and bulk density, as well as other parameters, including total soluble solids, total and reducing sugars, water absorption capacity, and swell index, which resulted in a higher consumer preference. Chromatography analysis confirmed the presence of polyphenols, organic acids, free sugars, and short-chain fatty acids, different from those of the control. The beverage was confirmed to be a probiotic using microbiological assessment with an effective probiotic count of 12 ± 1.24 (LPDB) and 13 ± 2.12 log CFU/mL (LPMB); also showing viability through harsh gut conditions like pH, lysozyme, and bile salts. The control (NPB) showed lower total phenolic and flavonoid content, compared to its fermented counterparts (LPDB, LPMB). In vitro analysis showed a significant increase in antioxidant potential illustrated through DPPH, ABTS, ABTS, anti-inflammation through NO assay, free radical scavenging through hydroxyl and superoxide radical scavenging, in probiotic beverages (LPDB, LPMB) than control (NPB). The probiotic beverage was studied for its oxidative stress alleviation through an in vivo model using *C. elegans,* showing enhanced health span parameters like locomotion and lower lipofuscin accumulation, better tolerance to thermal and oxidative stress. Fluorescence using DCF-DA showed decreased levels of ROS in probiotic beverage-treated nematodes, followed by non-probiotic beverage (NPB), with higher fluorescence intensity in the control (M9 buffer). Probiotic beverages (LPDB, LPMB) and non-fermented beverages (NPBs) of the study showed to increase the lifespan of *C. elegans*. Probiotic fermentation significantly enhanced antioxidant defenses and reduced oxidative stress markers in *C. elegans*, with LPDB and LPMB treatments markedly improving SOD and GSH levels while lowering CAT inhibition and MDA, similar to the effects of metformin. Overall, post-fermentation enhanced metabolites and bioactives, increasing health span and lifespan, underscoring the potential of pearl millet-based probiotic beverage in addressing longevity and age-related concerns by targeting oxidative stress and inflammation, also making it easier to target molecular and cellular aging biomarkers. Future studies include evaluating the food product in human clinical models, specifically to understand the impact on gut microbiota composition, systemic inflammation markers, and oxidative stress parameters. Also, understanding its efficacy across different population cohorts could broaden the scope of applicability in personalized nutrition strategies. The fermented millet-based probiotic beverage presents a significant promise as a sustainable, health-promoting food product that may contribute to the prevention and management of oxidative stress inflammation-related aging conditions.

## Figures and Tables

**Table 1:** Physicochemical parameters of fermented probiotic beverage.

Parameters	NPB	LPDB	LPMB
Titratable acidity (g/L)	2.36 ± 0.17 ^b^	6.23 ± 0.08 ^a^	6.49 ± 0.29 ^a^
pH	7.53 ± 0.07 ^a^	5.17 ± 0.67 ^b^	4.15 ± 0.18 ^c^
TSS (°B)	14.83 ± 0.25 ^a^	10.09 ± 0.31 ^b^	9.8 ± 0.28 ^c^
Bulk density (g/cm^3^)	0.58 ± 0.13 ^b^	0.86 ± 0.01 ^a^	0.84 ± 0.03 ^a^
Viscosity (cP)	856.00 ± 22.06 ^a^	590.00 ± 22.54 ^b^	577.00 ± 20.13 ^b^
Color analysis			
L*	29.96 ± 0.06 ^b^	30.58 ± 0.02 ^b^	32.45 ± 0.02 ^a^
a*	11.68 ± 0.04 ^a^	11.21 ± 0.05 ^b^	11.69 ± 0.05 ^a^
b*	17.66 ± 0.05 ^a^	17.67 ± 0.04 ^a^	17.98 ± 0.03 ^a^
ΔE	70.74 ± 0.07 ^a^	70.07 ± 0.03 ^a^	68.45 ± 0.04 ^b^
Reducing sugar (mg/100 mL WB)	7.59 ± 0.14 ^a^	3.20 ± 0.14 ^b^	3.18 ± 0.03 ^b^
Total sugar (mg/100 mL WB)	12.24 ± 0.72 ^a^	4.78 ± 0.19 ^b^	4.44 ± 0.21 ^b^

[NPB—Non-probiotic beverage; LPDB—*L. plantarum* DHCU70 probiotic beverage; LPMB—*L. plantarum* MCC 5231 probiotic beverage]. All of the values are mean ± standard error of the mean (SEM). Mean values with the same superscript letters are not significantly different, whereas those with the different superscript letters are significantly (*p* < 0.05) different, as judged by Duncan’s multiple range test.

**Table 2:** Fermentation metabolites in beverages.

Metabolite	NPB (Control)	LPDB	LPMB
Acetate	Not detected	Detected	Detected
Propionate	Not detected	Detected	Detected
Iso-butyrate	Not detected	Detected	Detected
Valerate	Not detected	Detected	Detected
Iso-propionate	Not detected	Detected	Detected
Methyl-propionate	Not detected	Detected	Detected
Vanillic acid	Detected	Not detected	Not detected
Catechin	Detected	Not detected	Not detected
Kaemferol	Detected	Not detected	Not detected
Rutin	Detected	Not detected	Not detected
Gallic acid	Not detected	Detected	Detected
Epigallocatechin gallic acid	Not detected	Detected	Detected
Di-hydroferulic acid	Not detected	Detected	Detected
Quercitin	Not detected	Detected	Detected
Caffeic acid	Not detected	Detected	Detected
4-vinyl guaiacol	Not detected	Detected	Detected

[NPB—non-probiotic beverage; LPDB—*L. plantarum* DHCU70 probiotic beverage; LPMB—*L. plantarum* MCC 5231 probiotic beverage].

**Table 3:** Microbiological parameters.

Parameter	Initial (0 h)	LPDB (12 h)	LPMB (12 h)
Viability	9.0 ± 0.00 ^b^	8.8 ± 0.07 ^c^	9.2 ± 0.04 ^a^
Resistance to pH (pH 2)	9.0 ± 0.00 ^a^	8.5 ± 0.15 ^b^	8.1 ± 0.17 ^c^
Resistance to bile salts (0.3% *w*/*v*)	9.0 ± 0.00 ^a^	8.6 ± 0.11 ^b^	8.4 ± 0.10 ^c^
Resistance to lysozyme (0.1% *v*/*v*)	9.0 ± 0.00 ^a^	8.3 ± 0.24 ^c^	8.7 ± 0.09 ^b^

[LPDB—*L. plantarum* DHCU70; LPMB—*L. plantarum* MCC 5231.] All of the values are mean ± standard error of the mean (SEM). Mean values with the same superscript letters are not significantly different, whereas those with the different superscript letters are significantly (*p* < 0.05) different, as judged by Duncan’s multiple range test.

**Table 4:** In vivo antioxidant enzyme assay.

Parameter/Groups	Control	NPB	LPDB	LPMB	Metformin
CAT inhibition (%)	23.49 ± 3.10 ^a^	11.37 ± 4.32 ^b^	8.11 ± 2.52 ^c^	8.67 ± 1.95 ^c^	7.65 ± 1.71 ^e^
SOD (U/mg protein)	0.52 ± 0.13 ^d^	0.83 ± 0.08 ^c^	1.53 ± 0.24 ^a^	1.38 ± 0.44 ^b^	1.64 ± 0.23 ^a^
GSH (nmol/mg protein)	12,620 ± 17.11 ^e^	13,952 ± 21.14 ^d^	16,875 ± 26.91 ^a^	15,067 ± 23.63 ^c^	15,383 ± 22.41 ^b^
MDA (nmol/mg protein)	0.89 ± 0.04 ^a^	0.76 ± 0.07 ^b^	0.65 ± 0.03 ^c^	0.61 ± 0.05 ^c^	0.49 ± 0.07 ^d^

[CAT—Catalase; SOD—Superoxide dismutase; GSH—Glutathione; MDA—Malondialdehyde; NPB—Non-probiotic beverage; LPDB—*L. plantarum* DHCU70 probiotic beverage; LPMB—*L. plantarum* MCC 5231 probiotic beverage.] Values are mean ± SD from three independent biological replicates (n ≈ 300 worms per group). Mean values with the same superscript letters are not significantly different, whereas those with the different superscript letters are significantly (*p* < 0.05) different, as judged by Duncan’s multiple range test. One-way ANOVA (F-statistic, df, *p*-value, η^2^ reported) followed by Duncan’s multiple range test (adjusted for multiple comparisons) was used for group comparisons. Normality (Shapiro–Wilk) and homogeneity of variance (Levene’s) were confirmed prior to ANOVA.

## Data Availability

The original contributions presented in the study are included in the article/Supplementary Material, further inquiries can be directed to the corresponding author.

## Supplementary Materials

The following supporting information can be downloaded at: https://www.mdpi.com/article/10.3390/foods14203460/s1. Figure S1. Polyphenolic compounds in A. NPB (Non-probiotic beverage) B. LPDB (*L. plantarum* DHCU70 probiotic beverage) C. LPMB (*L. plantarum* MCC 5231 probiotic beverage [a. Vanillic acid b. Catechin c. Kaemferol d. Rutin e. Gallic acid f. Epigallocatechin gallic acid g. Di-hydroferulic acid h. Quercitin i. Caffeic acid j. 4-vinyl guaiacol]. Figure S2. Short chain fatty acid profiling in a. LPDB (*L. plantarum* DHCU70 probiotic beverage) b. LPMB (*L. plantarum* MCC 5231 probiotic beverage). Figure S3. Organic acid profiling in a. NPB (Non-probiotic beverage) b. LPDB (L. plantarum DHCU70 probiotic beverage) c. LPMB (*L. plantarum* MCC 5231 probiotic beverage. Figure S4. Free saccharides profiling in a. NPB (Non-probiotic beverage) b. LPDB (*L. plantarum* DHCU70 probiotic beverage) c. LPMB (*L. plantarum* MCC 5231 probiotic beverage. Table S1. Effects of test compound concentrations on survivability. Table S2: Raw data of lifespan.
