# Supplementary material for: Physicochemical and Functional Characterization of Pearl Millet-Based Probiotic Beverage for Antiaging Potential in Caenorhabditis elegans
Source: Foods. 2025 Oct 10;14(20):3460. doi: 10.3390/foods14203460 (PMC12562646; doi:10.3390/foods14203460)
Supplement: Supplementary file 1 [file foods-14-03460-s001.zip › foods-3881191-supplementary.pdf]

SUPPLEMENTARY

I. Compositional analysis

1. Identification of polyphenols

For identifying polyphenols in ethanolic extracts of NPB and PB, HPLC system equipped with an LC-10AS pump and an SPD-M10A photodiode array detector, (Shimadzu, Japan) was employed. A Shodex C18-4E column (250 × 4.6 mm, 5 µm) was maintained at 30 °C. For the gradient mobile phase, pre-filtered (0.45 µm filter) (MF-Millipore, Bangalore, India) 0.1% acetic acid and HPLC grade acetonitrile were de-aerated and utilised. In each 20 min run, 5 µl of a diluted millet extract was injected. Photodiode array detector analysis was performed at 280 nm with a flow rate of 1 ml/min (Salar et al., 2016).

2. Identification of organic acids and free saccharides

Organic acids and free saccharides were identified through HPLC according to da Costa et al., (2016). The chromatographic setup included an LC-20AT pump along with a SPD-M10A diode array detector, which was connected in series with a RID-10A refractive index detector (Shimadzu, Kyoto, Japan). Carbohydrates and organic acids were separated using an Aminex Shodex C18-4E column (250 × 4.6 mm, 5 µm) and a pH range of 1–3. The separation was performed with a mobile phase of 3 mmol L−1 sulfuric acid (pH 2.35±0.02) under isocratic conditions. For each injection, 20 µL aliquots were processed at a flow rate of 0.5 mL min−1, at a constant column temperature of 60 °C. The total analysis time was 20 minutes, The detection wavelength for organic acids was set to 210 nm.

3. Identification of short chain fatty acids

30 mg of samples were mixed with 300 µl pure water and homogenized for 20 s under 6500 rpm for three times then incubated at 4 °C with shaking for 30 min, followed by centrifugation for 30 min at 13,000×*g*. 100 µl of supernatant was transferred into a new 0.6 mL microtube pre-added with 10 µl of 5 M HCl to bring the pH. The acidified samples were extracted by adding 100 µl of anhydrous DE (1:1, v/v), vortexed and incubated on ice for 5 min, and then centrifuged for 5 min at 10,000×*g*. The DE layer (containing SCFAs) was transferred to a new microtube containing anhydrous Na_2SO_4 (to remove the residual water). GC/MS analysis was performed by a 7890B gas chromatograph/5977 mass selective detector (Agilent Technologies, Santa Clara, CA, USA) with a HB-5 ms capillary column (30 m × 0.25 mm × 0.25 µm film thickness) (Agilent Technologies). The injector, ion source, quadrupole, and the GC/MS interface temperature were 260, 230, 150, and 280 °C, respectively. The flow rate of helium

carrier gas was kept at 1 mL/min. 1 µl of derivatized sample was injected with a 3 min of solvent delay time . The initial column temperature was 40 °C and held 2 min, ramped to 150 °C at the rate of 15 °C/min and held 1 min, and then finally increased to 300 °C at the rate of 30 °C/min and kept at this temperature for 5 min. The ionization was carried out in the electron impact (EI) mode at 70 eV (Zhang et al. 2019).

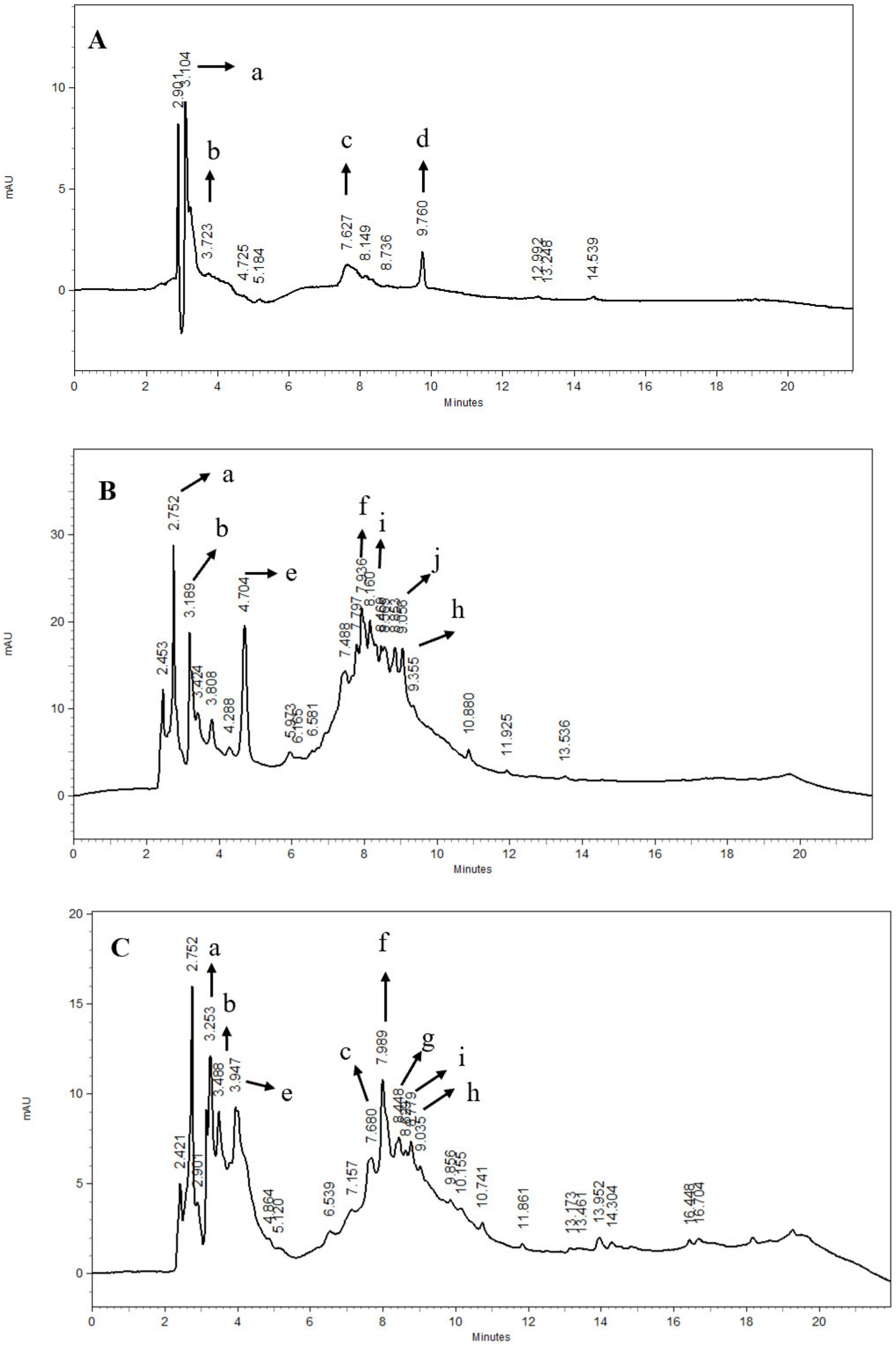

Figure S1. Polyphenolic compounds in **A.** NPB (Non-probiotic beverage) **B.** LPDB (*L. plantarum* DHCU70 probiotic beverage) **C.** LPMB (*L. plantarum* MCC 5231 probiotic beverage [**a.** Vanillic acid **b.** Catechin **c.** Kaemferol **d.** Rutin **e.** Gallic acid **f.** Epigallocatechin gallic acid **g.** Di-hydroferulic acid **h.** Quercitin **i.** Caffeic acid **j.** 4-vinyl guaiacol].

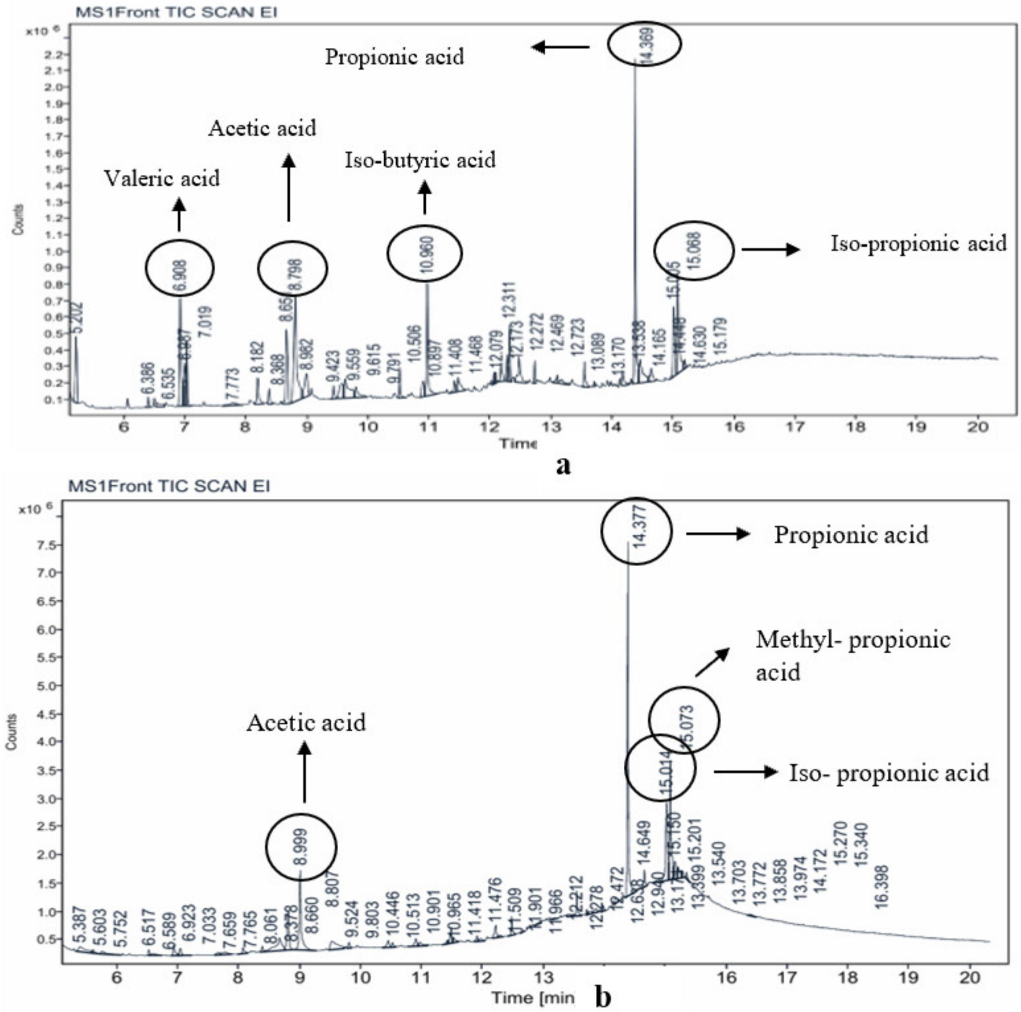

Figure S2. Short chain fatty acid profiling in **a.** LPDB (*L. plantarum* DHCU70 probiotic beverage) **b.** LPMB (*L. plantarum* MCC 5231 probiotic beverage).

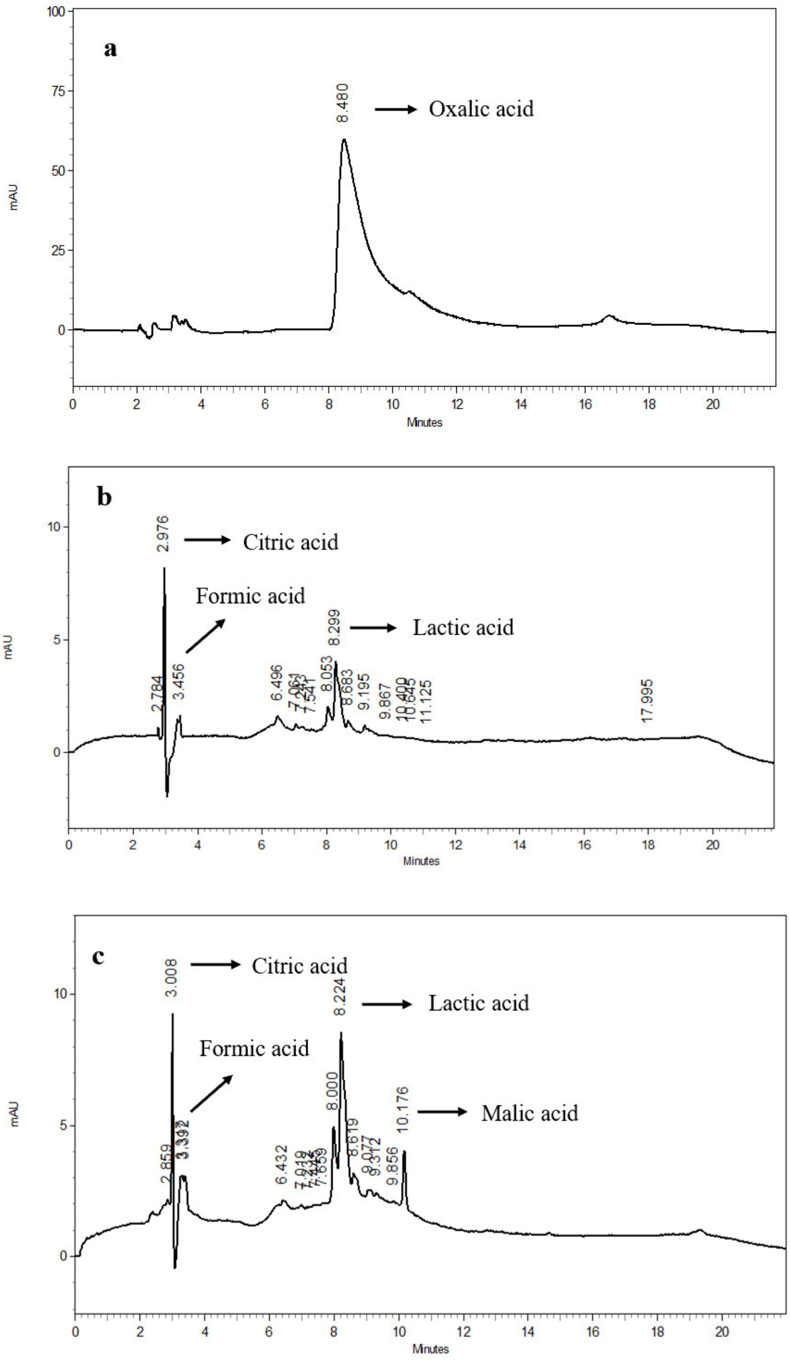

Figure S3. Organic acid profiling in **a.** NPB (Non-probiotic beverage) **b.** LPDB (*L. plantarum* DHCU70 probiotic beverage) **c.** LPMB (*L. plantarum* MCC 5231 probiotic beverage.

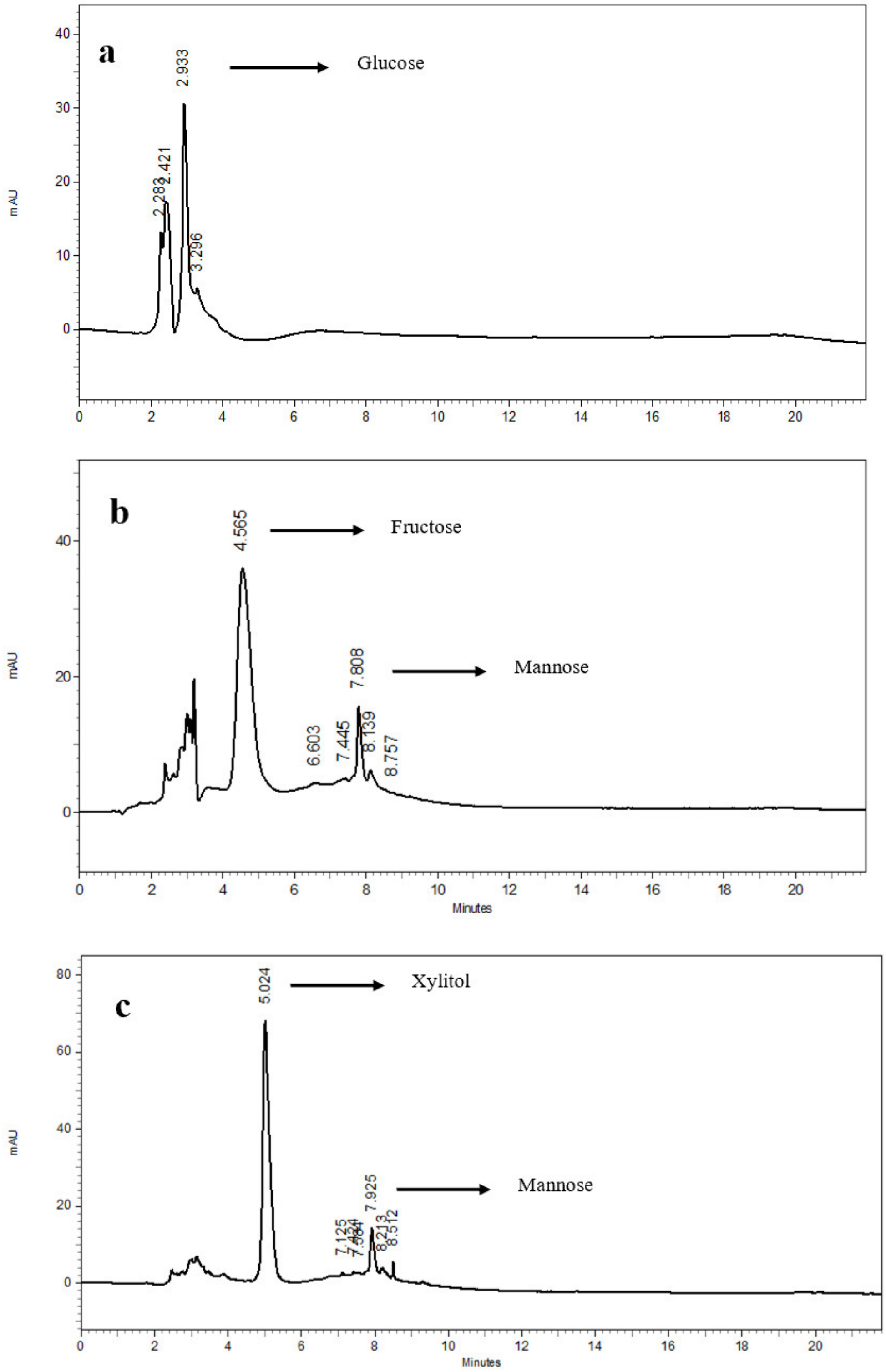

Figure S4. Free saccharides profiling in **a.** NPB (Non-probiotic beverage) **b.** LPDB (*L. plantarum* DHCU70 probiotic beverage) **c.** LPMB (*L. plantarum* MCC 5231 probiotic beverage.

II. Standard Operating Procedure (SOP) for Microbial Enumeration and Stress Resistance Assays

1. Scope

This SOP describes the procedures for serial dilutions, plating, colony counting, and calculation of CFU/mL and log CFU/mL for viability, lysozyme resistance, acid tolerance, and bile-salt resistance assays of probiotic cultures on MRS agar.

2. Materials

Probiotic culture in MRS broth, sterile phosphate-buffered saline (PBS), pH 7.2, MRS agar plates, lysozyme (0.1% v/v in PBS), HCl (for pH 2.0 adjustment), oxgall bile salts (0.3% w/v in MRS broth), sterile 1.5 mL microcentrifuge tubes, sterile 15 mL conical tubes, pipettes and sterile tips (10–1,000 µL range), anaerobic incubation jar or chamber, 37 °C incubator, vortex mixer, benchtop centrifuge, marker and plate racks.

3. Preparation

3.1. Grow starter culture

- Inoculate 10 mL MRS broth with a single colony; incubate anaerobically at 37 °C for 16 h.
- Centrifuge 5,000 × g, 10 min, 4 °C; discard supernatant.
- Wash cell pellet twice with 10 mL sterile PBS; resuspend in 10 mL PBS.

3.2. Prepare stress solutions

- Lysozyme solution: dissolve 0.1% (v/v) lysozyme in PBS.
- Acid solution: adjust PBS to pH 2.0 with concentrated HCl.
- Bile solution: dissolve 0.3% (w/v) oxgall in MRS broth.

4. Serial Dilution and Plating

4.1. Label tubes for 10^{-1} through 10^{-10} dilutions.
4.2. Add 900 µL PBS to each tube.
4.3. Pipette 100 µL of resuspended culture into 900 µL PBS (10^{-1}); vortex 10 s.
4.4. Repeat serial transfers to 10^{-10}.
4.5. For each condition and time point, plate 100 µL of the 10^{-7} dilution on MRS agar in triplicate; spread evenly.

5. Incubation

Place plates in an anaerobic jar or chamber and incubate at 37 °C for 12 h.

6. Colony Counting

6.1. After incubation, count discrete colonies on each plate.
6.2. Record counts as Plate 1, Plate 2, Plate 3 for each condition.

7. Data Calculation

7.1. **CFU/mL**

CFU/mL=colonies×10^7/0.1 mLCFU/mL=0.1mLcolonies×10^7

7.2. **Logarithmic Transformation**

log10(CFU/mL)(base-10 log)log10(CFU/mL)(base-10 log)

7.3. Calculate mean and standard error of the mean (SEM = SD/√n; n = 3).

8. Stress Resistance Assays

Perform stress treatments immediately after washing pellet:

8.1. Lysozyme: incubate 0.1% lysozyme solution, 37 °C, 1 h.

8.2.Acid: incubate pH 2.0 PBS, 37 °C, 3 h.
8.3.Bile: resuspend in 0.3% oxgall MRS broth, anaerobic, 37 °C, 12 h.
Proceed with serial dilution, plating, and counting as above.

9. Quality Control

- Include uninoculated PBS controls at each dilution to check sterility.
- Validate colony morphology on MRS agar for target probiotic strain.Repeat any outlier counts that vary by > 20% from triplicate mean.

CONSENT FORM

DESCRIPTION:

The sensory evaluation of probiotic millet beverage will be assessed in terms of color, texture, aroma, and taste flavor characteristics. The scorecards used will be tabulated and data collected will be kept for three years.

CONFIDENTIALITY:

Participant's names will not be on the scorecards and the name and any other identifiers will be kept in a locked file that is only accessible to the PI and his/her research associates. Any information from this study that may be published and will not identify the participants/panelists.

BENEFITS:

The results of this study may benefit food processors by having a standardized method to evaluate food products. There will be no direct benefit to participants /panelists from participating in this study.

RISKS:

It is possible that some fermented_samples may be slightly sour or off-flavor and may cause throat or mouth irritation. However, there are no other known risks to the participants/panelists.

CONTACT PEOPLE:

If you have any questions about this research, please contact the Principal Investigator. His/her contact details are given below. If you have any questions about your rights as a research subject, please contact the Office of the Traditional Food and Sensory Science Department, Central Food Technological Research Institute at 0821-2515842.

VOLUNTARY NATURE OF PARTICIPATION:

Your participation in this study is voluntary. If you don't wish to participate or would like to end your participation in this study, there will be no penalty or loss of benefits to you to which you are otherwise entitled. In other words, you are free to make your own choice about being in this study or not and may quit at any time without penalty.

SIGNATURE:

Your signature on this consent form indicates that you fully understand the above study, what is being asked of you in this study, and that you are signing this voluntarily. If you have any questions about this study, please feel free to ask them now or at any time throughout the study.

Please indicate any known allergies to food:

Participant

Name: _________________________

Signature with date_______________________

Thank you for taking the time to become a part of the endeavour

Dr. Muthukumar S P, Department of Biochemistry, CSIR-CFTRI

Acute toxicity studies

The toxicity tests in accordance with Qin et al. (2022) with some modifications. The L4-stage synchronized populations were washed off twice with M9 buffer. Concentrations observed for optimal survivability in nematodes for beverages and metformin from 100-500 µg with M9 as a negative control (Qin et al., 2022) for 24 h.

Table S1: Effects of test compound concentrations on survivability

**Extract**	**Concentration (µg)**	**Survival Rate (%)**
**NPB**	100	96
	150	95
	200	95
	250	62
	300	22
	350	18
	400	9
	450	4
**LPDB**	100	97
	150	96
	200	95
	250	87
	300	40
	350	22
	400	13
	450	9
**LPMB**	100	97
	150	97
	200	96
	250	85
	300	37
	350	19
	400	11
	450	3
**Metformin**	100	95
	150	83
	200	68
	250	48
	300	33
	350	12
	400	6

The results are presented as mean of % viability. Significance differences between treatment and positive groups found ($p>0.05$).

Table S2: Raw data of lifespan

Time (days)	Control	NPB	LPDB	LPMB	Metformin
0	120±3.00	120±2.00	120±2.00	120±3.00	120±2.00
4	103±16.00	105±12.00	108±11.00	111±11.00	118±12.00
8	94±11.00	98±15.00	101±12.00	98±10.00	113±10.00
12	73±12.00	84±15.00	87±7.00	88±9.00	107±9.00
16	55±9.00	73±10.00	76±12.00	74±11.00	100±14.00
20	38±8.00	61±7.00	66±9.00	64±9.00	94±7.00
24	24±6.00	36±9.00	41±7.00	50±9.00	62±11.00
28	0±0.00	0±0.00	16±4.00	21±7.00	30±8.00
**Survival probabilities**					
0	1	1	1	1	1
4	0.8583	0.875	0.9	0.925	0.9833
8	0.7833	0.8167	0.8417	0.8167	0.9417
12	0.6083	0.7	0.725	0.7333	0.8917
16	0.4583	0.6083	0.6333	0.6167	0.8333
20	0.3167	0.5083	0.55	0.5333	0.7833
24	0.2	0.3	0.3417	0.4167	0.5167
28	0	0	0.1333	0.175	0.25

All of the values are mean ± standard error of the mean (SEM). The reported values represent the mean of three independent experiments, each consisting of three plates with 40 worms per plate (total n = 120 worms per experiment).

Kaplan-Meier Estimator for Survival Probability $S(t)=\prod_{t_i\leq t}(1-\frac{d_i}{n_i})$

where:

$S(t)$ = Probability of survival at time ‘t’

d_i= Number of deaths at time t_i

n_i = Number alive just before t_i

References

1. da Costa, M.P., da Silva Frasao, B., da Costa Lima, B.R.C., Rodrigues, B.L. and Junior, C.A.C., 2016. Simultaneous analysis of carbohydrates and organic acids by HPLC-DAD-RI for monitoring goat's milk yogurts fermentation. *Talanta*, *152*, pp.162-170.

2. Qin, Y., Chen, F., Tang, Z., Ren, H., Wang, Q., Shen, N., Lin, W., Xiao, Y., Yuan, M., Chen, H. and Bu, T., 2022. Ligusticum chuanxiong Hort as a medicinal and edible plant foods: Antioxidant, anti-aging and neuroprotective properties in Caenorhabditis elegans. *Frontiers in Pharmacology*, *13*, p.1049890.

3. Salar, R.K., Purewal, S.S. and Sandhu, K.S., 2017. Fermented pearl millet (Pennisetum glaucum) with in vitro DNA damage protection activity, bioactive compounds and antioxidant potential. *Food Research International*, *100*, pp.204-210.

4. Singhal, N., Singh, N.S., Mohanty, S., Kumar, M. and Virdi, J.S., 2021. Rhizospheric Lactobacillus plantarum (Lactiplantibacillus plantarum) strains exhibit bile salt hydrolysis, hypocholestrolemic and probiotic capabilities in vitro. *Scientific Reports*, *11*(1), p.15288.

5. Zhang, S., Wang, H. and Zhu, M.J., 2019. A sensitive GC/MS detection method for analyzing microbial metabolites short chain fatty acids in fecal and serum samples. *Talanta*, *196*, pp.249-254.
